# Annotation, submission and screening of repetitive elements in Repbase: RepbaseSubmitter and Censor

**DOI:** 10.1186/1471-2105-7-474

**Published:** 2006-10-25

**Authors:** Oleksiy Kohany, Andrew J Gentles, Lukasz Hankus, Jerzy Jurka

**Affiliations:** 1 Genetic Information Research Institute, 1925 Landings Drive, Mountain View, CA 94043, USA; 2 School of Medicine, Stanford University Stanford, CA 94301, USA

## Abstract

**Background:**

Repbase is a reference database of eukaryotic repetitive DNA, which includes prototypic sequences of repeats and basic information described in annotations. Updating and maintenance of the database requires specialized tools, which we have created and made available for use with Repbase, and which may be useful as a template for other curated databases.

**Results:**

We describe the software tools RepbaseSubmitter and Censor, which are designed to facilitate updating and screening the content of Repbase. RepbaseSubmitter is a java-based interface for formatting and annotating Repbase entries. It eliminates many common formatting errors, and automates actions such as calculation of sequence lengths and composition, thus facilitating curation of Repbase sequences. In addition, it has several features for predicting protein coding regions in sequences; searching and including Pubmed references in Repbase entries; and searching the NCBI taxonomy database for correct inclusion of species information and taxonomic position. Censor is a tool to rapidly identify repetitive elements by comparison to known repeats. It uses WU-BLAST for speed and sensitivity, and can conduct DNA-DNA, DNA-protein, or translated DNA-translated DNA searches of genomic sequence. Defragmented output includes a map of repeats present in the query sequence, with the options to report masked query sequence(s), repeat sequences found in the query, and alignments.

**Conclusion:**

Censor and RepbaseSubmitter are available as both web-based services and downloadable versions. They can be found at  (RepbaseSubmitter) and  (Censor).

## Background

Repbase is the most widely used database of transposable elements, with ~5800 entries as of October 2006, representing over 40 superfamilies of DNA transposons, LTR and non-LTR retrotransposons, and endogenous retroviruses [1]. The current version of Repbase is based on a flexible and extensible relational database schema implemented in mySQL. Ongoing large-scale sequencing of eukaryotic genomes has resulted in a rapid increase in the rate at which new transposable elements are discovered. Rather than relying on error-prone automated processing, the philosophy behind Repbase has been to incorporate a significant amount of manual curation into the database. However, the increasing number of sequences to be annotated and entered led us to develop a standardized submission interface that external users can use to provide information on their sequences, with a minimum of subsequent reformatting being necessary.

Repbase is primarily being used for screening and annotation of genomic DNA. Censor was the first program for Repbase-based repeat detection and masking, originally released in 1994 and later published [2]. Its major drawback was inefficient implementation of the Smith-Waterman algorithm and, therefore, the publicly accessible Censor server ran exclusively on specialized Paracel hardware. In the meantime, other programs, notably RepeatMasker [3,4], and *blaster *[5] became available. RepeatMasker uses a customized version of the Repbase library that can sometimes have significant differences from the original Repbase submission. Furthermore, Censor can be used to search DNA sequences against a library of proteins, or translated nucleotide sequences.

Manual curation of databases has both advantages and drawbacks compared to automated processing. Automatic annotation has the significant advantage of much higher potential throughput, freedom from user error, and elimination of unintended bias in the processing. On the other hand, it is hard to anticipate every contingency in, for example, correct reconstruction of consensus sequences. A particular problem with automated reconstruction of transposable elements is over-fragmentation, where algorithms do not correctly assemble related parts of an element into a complete consensus. The principal source of mistakes in manual curation is user error in entering data. All complex data such as taxonomy, literature references, transposable element classifications are potentially problematic, since simple misspellings can render a database entry unretrievable based on exact string-based searches.

For these reasons, we have chosen to adopt a hybrid approach: keeping the positive aspects of manual curation, while attempting to eliminate the most common sources of user-supplied errors, by automating the import and annotation of complex, but well-defined information including taxonomic information, referencing, etc. The purpose of RepbaseSubmitter is to provide an easy to use interface that permits flexibility in annotation, while at the same time reducing the scope for mistakes in the manual curation process.

## Implementation

### RepbaseSubmitter

RepbaseSubmitter is implemented in Java (requires Java Virtual Machine version 1.5 or above). The interface is structured around six data entry pages, together with an initialization page for creating a new entry, and a final submission page for performing checks and submitting to Repbase. New entries are not directly entered into Repbase, but are submitted to a review database for editorial approval and additional curation.

### Censor

The new version of Censor described here uses an unaltered version of Repbase (as well as user-supplied libraries if desired), and is composed of Perl and C++ modules for identification of both interspersed and tandem repeats using similarity searches. Censor analyzes DNA/RNA sequences for repeats and provides a description of repetitive elements from their Repbase Update annotation [1]. Censor can optionally use either WU-BLAST or NCBI BLAST as its search engine, and can perform both direct NA-NA searches, as well as any combination of protein-NA or translated NA searches using the appropriate BLAST modules.

The downloadable version of Censor can be installed on virtually any UNIX system (including Mac OSX) with Perl and a C++ compiler, that has WU-BLAST or NCBI BLAST installed. It can also utilize symmetric multiprocessor machines. Censor uses BLAST to detect similarity between repeat libraries and nucleic acid (NA) or protein sequences. For simple NA-NA searches, BLASTN is used (the default). For more sensitive detection of distantly-related protein coding sequences, a six-frame search (using TBLASTX) or protein-NA search (TBLASTN) is available. Censor offers three sensitivity modes: normal, rough and sensitive, each offering a different balance of sensitivity and speed. The difference in performance is determined by the BLAST search parameters (see Additional file 1). Censor automatically determines the type (NA or protein) of input sequences by calculating base composition, and calls the appropriate BLAST program, although this behavior can be overridden as described below. Censor relies on some standard UNIX system commands. For that reason a Unix/Linux operating system is required. Censor requires Perl to work, which is standard on most UNIX systems. WU-BLAST (recommended) or NCBI-BLAST is required to perform searches. If the BLAST installation directory is on the user's path, the configuration script will automatically detect it and assign corresponding variables. Otherwise, users must manually edit the header of Censor's main script to provide this information. GCC or another C++ compiler, and "make' utility, are required to build the Censor distribution.

## Results and discussion

### RepbaseSubmitter

At all stages of data entry using the submission interface, required fields are indicated by boxes highlighted in red. Although the data entry forms can be accessed in any order, if required information is omitted, the program will not allow the user to proceed until it has been entered. The entry forms of RepbaseSubmitter, and the main information that can be entered through them, are summarized in Table 1. The Initialization (**Select**) page allows the user to begin creation of a new Repbase sequence by loading data from a pre-existing file, or by starting with a completely blank template. After this initial selection, the **Summary **data entry page is displayed. The primary fields required for creation of a new entry include a Repbase accession number. The format of accession numbers is not fixed, and is user-defined; however, it must be unique. This Repbase identifier can be considered analogous to a HUGO gene name, rather than an abstract database entity such as a Genbank accession number. There is no currently accepted standard of assigning of names to transposable elements. However, this topic was the subject of a recent special working group (Asilomar Conference on "Genomic Impact of Transposable Elements", Asilomar, USA, Mar 31 – Apr 4, 2006). The Summary page also requires a description of the sequence being submitted. Ideally this is a succinct outline of the sequence type and nature, for example "L1-1_MD: a young L1 element from Monodelphis domestica – consensus sequence". A comments section is also available for a more detailed description of the sequence, and is not limited in scope. Examples of such information might include number of copies of the sequence in a genome; age distribution of transposable elements (e.g. the mean similarity of copies to the consensus sequence); relationship of this sequence to other transposable elements that may be of interest; etc. Finally, it is possible to specify free-form keywords which provide pertinent information specific to this sequence. Repbase entries can be searched by keyword, so a user may wish to specify information such as characterization of protein coding domains present in the sequence (e.g. reverse transcriptase, endonuclease). The keyword field is also used internally by Repbase to indicate links to corresponding RepeatMasker library entries. The Summary page also notes the IP address of the computer submitting the data to Repbase – this is not user-editable.

**Table 1:** Data entry pages in RepbaseSubmitter.

**Data Entry Form**	**Purpose**
Select	Initialization page
Summary	Specification of entry Accession, Keywords, Definition, Comments
Sequence	Entry of sequence, calculation of DNA content and lengths
Organism	Source organism/taxonomy; classification based on current Repbase structure
Protein	Specification of coding regions: prediction of ORFs, annotation on DNA sequence, comments describing protein features/functions
References	Relevant references to primary literature or databases (Repbase or external such as Genbank, EMBL)
Release	Repbase release, relevant database accessions; consensus references
Submission	Display of final version prior to submission, perform final checks, submit to relational database for review

The seven main forms presented by RepbaseSubmitter are listed with their title, and a summary of the information which can be entered.

The **Organism **entry page ensures that correct taxonomy of entries is maintained; both at the level of species, and for classes of repeat element. As species name is typed, RepbaseSubmitter dynamically searches the NCBI taxonomy tree [6] and lists matching entries. The species can be selected from the list as soon as the correct one appears, or can be typed fully – the more of the species name that is typed, the narrower the list presented. Once a specific species has been selected, the interface pulls the correct taxonomic classification from the NCBI Taxonomy database, and enters this information in the relevant field. In addition, this section of the interface facilitates correct classification of transposable entries. The current classification scheme implemented in Repbase is given in Table 2, however the scheme is transparently extensible as new superfamilies of transposable element are identified. The status of the sequence as an autonomous or non-autonomous element can also be specified at this point. If non-autonomous, the corresponding mobilizing element may be indicated.

**Table 2:** Current Repbase schema for transposable element classification.

**Major Class**	**Superfamilies**
**DNA transposons**	Mariner, hAT, MuDR, EnSpm, piggyback, P, Merlin, Harbinger, Transib, Novosib, Mirage, Helitron, Polinton, Rehavkus
**LTR retrotransposons**	Gypsy, Copia, DIRS, BEL
**Endogenous retroviruses**	ERV1, ERV2, ERV3
**Non-LTR retrotransposons**	LINE1 (L1), RTE-1, CRE, CR1 (LINE3), I, Jockey, NeSL, R2, R4, Rex1, RandI, Penelope
**Caulimoviridae**	
**Simple repeat**	Satellites (SAT, MSAT)

Repbase currently recognizes over 40 superfamilies of transposable/repetitive element. The major classes and superfamilies are listed here. The underlying relational database structure of Repbase allows easy addition and modification of the classification scheme, based on currently accepted conventions.

The **Sequence **entry page is the simplest, and requires only the sequence data to be input. If a DNA or RNA sequence was loaded from file at the initial entry creation page, it will be displayed here. Otherwise, sequence data can be cut-and-pasted into the window. The base count and composition of the sequence is automatically updated and entered. Sequences can also be complemented, if it is determined that the other strand is more appropriate (for example, if it encodes proteins for autonomous elements).

**Figure 1:**
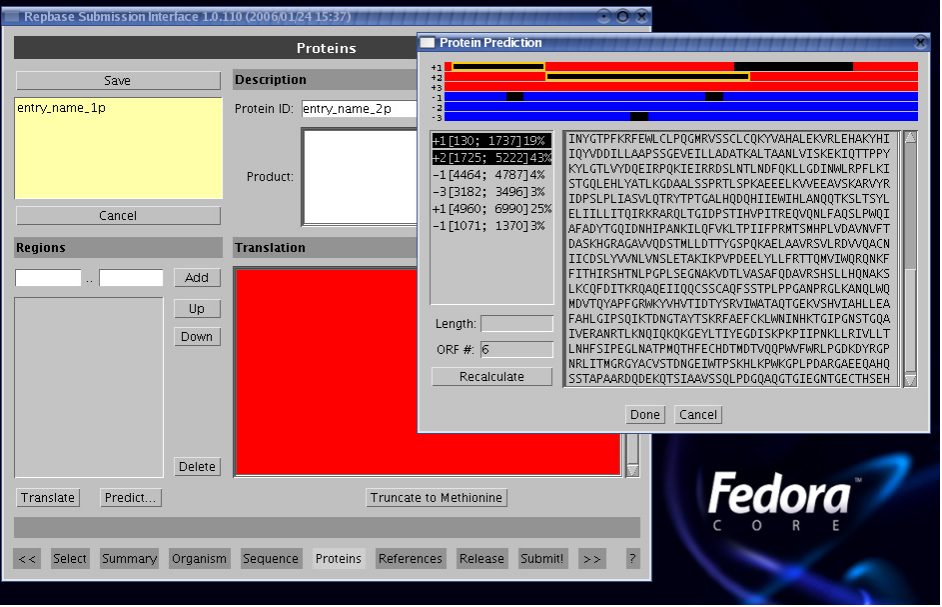
**Protein annotation entry form of RepbaseSubmitter**. The protein prediction sub-window is also shown, showing how ORFs can be predicted and merged into a predicted protein for annotation on the nucleotide sequence. The bottom of the main window shows access buttons for each entry page of the program. RepbaseSubmitter is written in java, and can run on any system with an installed Java Virtual Machine of version 1.5 or above.

Autonomous transposable elements encode proteins such as transposase, reverse transcriptase, endonucleases, etc. This information is often of interest to researchers using Repbase, and the **Proteins **interface (shown in Fig. 1) provides a convenient way for identification and annotation of open reading frames (ORFs) in the sequence. Multiple proteins can be specified for the same Repbase entry, and therefore it is necessary to supply a unique Repbase protein identifier. One is generated automatically for each ORF added – users may choose to specify their own identifier, but it must be unique in Repbase, and will be checked at the final stage before upload to the review database. A comment field is associated with each protein entry on a sequence. Coordinates of coding regions can be entered manually, and the corresponding region will be translated and entered as the coding sequence. However a useful feature of the Protein annotation page is the ability to predict ORFs. Upon selecting the "Predict" option on this page, the user is prompted to specify how many ORFs, *N*, are anticipated. The program will graphically display the *N *longest ORFs on all strands, along with their corresponding coordinates in the sequence. The user can select an ORF to add to the Repbase entry as a putative protein coding region; in addition, several fragments of ORFs can be merged together as one coding region if it is anticipated that they are part of the same protein. This is generally only recommended if resulting gaps are small. Finally, an option is provided to truncate a specified coding region to the first occurring Methionine.

An important feature of Repbase is the ability to supply references to appropriate scientific literature, or to other Repbase entries and other databases. The submission interface facilitates both types of referencing. References to scientific literature can be added manually i.e. by supplying authors, title, journal etc. in the normal manner; however, in this case entries are not automatically verified in any way. As an alternative, RepbaseSubmitter provides an "Import" option on the **References **entry page. This allows users to specify partial information such as author names, article title, journal name, and then search the NCBI Pubmed database [7]. A list of matching references is returned, and multiple selections can be made from this list and included in the Repbase entry. In this way, references to literature will correspond exactly to how they appear in Pubmed, which can substantially eliminate errors due to mistyping of reference information. In some cases, a particular reference may only apply to part of a sequence. This is often true if the sequence currently being entered is an extension of a previously-existing partial Repbase entry; or if the element being annotated combines information that has been reported fragmentarily in multiple locations. A reference may also be to another database such as Genbank or EMBL, or to another Repbase entry. In this case, the user needs to supply the author information manually. If the creation of this Repbase entry represents new work, the user will generally want to supply a title, and submit it to Repbase Reports. Entries already described in another publication should be directed to Repbase Update. Repbase Reports provides a medium for publication of novel transposable elements in an online journal form, so that the work may be referred to in other publications. Finally, the Reference page provides an option for "Free Text" references, for those cases which do not correspond to traditional journal references, or links to those databases specifically recognized by Repbase.

The **Release **and Accessions page summarizes the information supplied on the References page, primarily to allow selection of a primary reference for sequences which are consensi. Additionally, it is possible to specify a "creation date" for this Repbase entry (generally the current date); and a "last update" which will be the same as the creation data for a new sequence, but may be different if this is a refinement of a pre-existing Repbase element. This section is also the appropriate place to specify accession number(s) linking to other databases (Genbank etc.) – one accession number will be the *primary accession *for the sequence.

The last screen of the submission interface is for actual submission to the Repbase review database. The database entry as it will appear in native Repbase (EMBL) format is displayed, and may be saved to a file. Upon selecting "submit", the entry is checked for correct formatting, and basic consistency such as unique Repbase accession and sequence information; and is then entered into the mySQL database for approval

## Censor

### Pre-processing of data

Before performing each search, input data is checked and formatted. Censor automatically chops long sequences into smaller fragments to reduce BLAST memory requirements and to facilitate splitting of jobs on multiple processor machines. Base composition is calculated for query and database sequences, and based on the total percentage of ATCGN bases, Censor decides whether each sequence is nucleotide or protein. This information is used in automatic selection of the BLAST search program – BLASTN, BLASTP, BLASTX or TBLASTN. In order to run a translated versus translated search of nucleotide against nucleotide sequences, TBLASTX must be specified as the search program (otherwise BLASTN is used). By default, simple tandem repeats are masked using filter modules prior to similarity searching, to prevent false hits. Two approaches are available for dealing with simple repeats. The built-in BLAST filters, SEG and DUST, can be applied in initial sequence processing. However this prevents identification of simple repeats in the Censor output. Another approach is to mask them by first BLASTing the query sequence against a library of simple repeats, which is included with the Censor distribution. In this case simple repeats will be reported in the program's output. Both filtering functions can be disabled if required, but this is not recommended, since it can lead to a significant proportion of false hits between the query sequence and simple repeats that are internal parts of repetitive elements curated in Repbase. However, disabling *annotation *of simple repeats can lead to a significant decrease in overall processing time.

### Similarity searching

In the main search phase, Censor uses BLAST to compare the input sequence to annotated repetitive elements in Repbase, or a custom user-supplied library. There are two separately developed and maintained versions of BLAST available: WU-BLAST, copyrighted and maintained by Washington University [8], and a free version developed by NCBI [9]. Both versions have their advantages and disadvantages. WU-BLAST is faster than NCBI BLAST, and has more options, making it very flexible. However WU-BLAST requires licensing from commercial companies and academic users (this can be done online for the latter), while NCBI BLAST is free for all users. As a result, we created two versions of standalone Censor, with parameters optimized for each version of BLAST. A web-based Censor server is also available, which uses WU-BLAST solely. The default WU-BLAST parameters for Censor's "normal", "sensitive", and "rough" modes are described in the Supplementary Material (see Additional file 1). In addition, all BLAST parameters can be overridden by specification on the command line of standalone Censor. The query sequence is scanned against each library of repeats specified using Censor's "-lib" option, in the order in which they are listed. After processing each library, detected repeats are masked out from the query sequence before comparison to the next library.

### Post-processing and output

**Figure 2:**
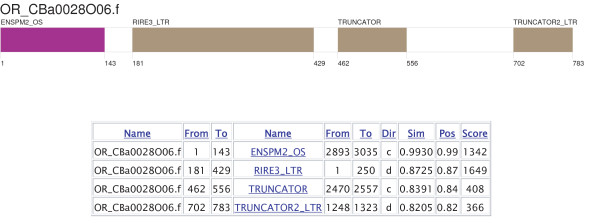
**Example of a repeat map, and graphical representation**. **Name **contains locus names of submitted query sequences (first column) and library sequences (fourth column). Repbase names are hyperlinked to their sequences in web-based Censor. **From/To **contains beginning/end positions of reported fragments on their corresponding sequence. **Dir **indicates orientation ('d' for direct, 'c' for complementary) of repeat fragment. Column **Sim **contains the similarity between 2 aligned fragments, calculated as described in the text. **Pos **is roughly the ratio of positive matches (bases that produce positive scores in the alignment matrix) to alignment length. This ratio is calculated the same way as we calculate similarity (see main text), with *positive_count *instead of *match_count*. This information is particularly useful for estimating the quality of protein alignments. **Score **is the alignment score obtained from BLAST.

Censor performs post-processing of BLAST output by removing overlaps and defragmenting detected repeats. The program reports positions of repetitive elements in ".map" files. Figure 2 shows an example of a repeat map. Many methods for evaluating the similarity between two or more homologous sequences exist [10-12]. In the case of transposable elements, even a large indel (insertion or deletion), which corresponds to any uninterrupted alignment gap, can reflect one event in evolution (transpositional insertion or excision) and should impact the value of similarity the same way unrelated to its length. The similarity values output in maps are therefore calculated as follows: *Sim = match_count/(alignment_length - query_gap_length - subject_gap_length + gap_count) *where: *match_count *= number of matching base positions in alignment; *alignment_length *= length of alignment, i.e. number of matches + number of mismatches + length of gaps; *query_gap_length *= total length of alignment gaps on submitted query sequence; *subject_gap_length *= total length of alignment gaps on library sequence; *gap_count *= number of uninterrupted alignment gaps of any length on either query or subject sequences. In addition to this measure, the Censor output incorporates an alternative similarity measure *Pos*, that is calculated on the basis of positive scores between aligned base pairs. This is typically higher than the previous similarity score, and may be more appropriate for protein alignments. Furthermore, Censor can produce pair-wise alignments of detected repeats using the SWAT algorithm [13]. For these, the similarity reported incorporates an affine gap penalty.

Maps include simple repeats unless the "-nosimple" option was specified. The web-based version of Censor provides a graphical representation of the map in SVG (Scalable Vector Graphics) format, with colour-coding of different repeat types. By default, Censor also produces a ".masked" file containing the original sequence with all detected repeats masked out; and a ".found" file containing the genomic sequence fragments that were detected as matching a known repeat. General information on the query sequence(s) and their repeat content is stored in ".tab" files.

Finally optional tasks are performed, including classification of repeats into subfamilies based on maximum similarity to consensus sequences. Currently the Censor distribution supports only classification of human ALU subfamilies. However other repeat families can be classified after an easy setup process that requires a list of consensus sequences and a hierarchy of subfamilies. A complete description of Censor parameters can be found in the program documentation. Details of BLAST parameters for the available sensitivity modes are given in the Supplementary Material (see Additional file 1).

## Conclusion

The resulting new package, RepbaseSubmitter, facilitates and automates many aspects of Repbase entry creation and maintenance. The program performs numerous checks on formatting of entries, and consistent entry of certain data fields; as well as ensuring that required data are provided.

## Availability and system requirements

Project name: Censor

Project home page: 

Operating system(s): Unix/Linux

Programming language: Perl, C++

License: GPL

Any restrictions to use by non-academics: None

Project name: RepbaseSubmitter

Project home page: 

Operating system(s): Any, with Java Virtual Machine 1.5 or above

Programming language: Java

Other requirements: Java 1.5 or higher

License: GPL

Any restrictions to use by non-academics: None

## Authors' contributions

OK wrote and developed software for Censor and RepbaseSubmitter. AG helped with debugging and feature addition of both programs, and wrote the manuscript. LH did the initial design and coding of Repbasesubmitter. JJ directed development of both programs as Principal Investigator. All authors contributed to and approved the final manuscript.

## Supplementary Material

Additional file 1 Supplementary Material A. Parameters supplied to WU-BLAST by Censor Click here for file
