# Supplementary material for: Annotation, submission and screening of repetitive elements in Repbase: RepbaseSubmitter and Censor
Source: BMC Bioinformatics. 2006 Oct 25;7:474. doi: 10.1186/1471-2105-7-474 (PMC1634758; doi:10.1186/1471-2105-7-474)
Supplement: Additional file 1 — Supplementary Material A. Parameters supplied to WU-BLAST by Censor [file 1471-2105-7-474-S1.doc]

**Supplementary Material A: Parameters supplied to WU-BLAST by Censor**

All programs are supplied the following arguments, described in the documentation for BLAST:

<BLAST program> DB QU -gi hspmax=0 gspmax=0 B=100000000 V=0 gapE2=0.001 -warnings

Additional command line parameters for each blast program for different sensitivity levels (norm, rough, sens) are listed below.

BLASTN:

-gapW=76 -wordmask=dust -gapall

plus:

|  | W | Q | R | S2 | gapS2 | S | X | gapX | wink | -matrix | Other |
| --- | --- | --- | --- | --- | --- | --- | --- | --- | --- | --- | --- |
| **norm** | 7 | 30 | 6 | 112 | 225 | 225 | 225 | 450 | - | 20p<CG>g^*^ |  |
| **rough** | 8 | 30 | 6 | 112 | 225 | 225 | 225 | 450 | 2 | 20p<CG>g | -hitdist=50 |
| **sens** | 6 | 27 | 6 | 110 | 220 | 220 | 220 | 440 | - | 25p<CG>g |  |

^*^In actual use, <CG> refers to G+C content of the query sequence. Censor incorporates substitution matrices that are optimized for sequences of different G+C content [3]. For examples matrix 20p39g is used for nucleotide sequences with average G+C content of 39%. The invocation of these matrices is done transparently to the user.

BLASTP, BLASTX, TBLASTN, TBLASTX:

-gapall E=0.05 -kap -wordmask=seg+xnu gapE2=0.001

plus:

|  | T | Other |
| --- | --- | --- |
| **norm** | 1000 |  |
| **rough** | 1000 | wink=2, hitdist=200 |
| **sens** | - |  |
